# The association between a body shape index and cardiorespiratory fitness among adolescents in rural China: a sex-specific cross-sectional study

**DOI:** 10.3389/fnut.2026.1871460

**Published:** 2026-08-14

**Authors:** Chaogong Zeng, Chengjun Chai, Jiali Xu, Mian Sha, Jian Xin

**Affiliations:** 1 Physical Education College, Shanghai University, Shanghai, China; 2 Physical Education College, Shangrao Normal University, Shangrao, China; 3 Party Committee Office, Gannan Medical University, Gannan, China

**Keywords:** 20-m shuttle run test, a body shape index, adolescents, cardiorespiratory fitness, rural areas

## Abstract

**Background:**

Cardiorespiratory fitness (CRF) among Chinese adolescents has been declining year by year, with the decline being particularly pronounced in rural areas. As a novel indicator of body composition, the a body shape index (ABSI) has rarely been examined in previous studies regarding its association with CRF among adolescents in rural areas. This study aims to guide the improvement and enhancement of CRF among adolescents in rural China.

**Methods:**

Using a stratified random cluster sampling method, we conducted a cross-sectional assessment of height, weight, waist circumference, and 20-m shuttle run test (20-m SRT) performance among 18,948 adolescents aged 6–17 years in rural areas of China. We analyzed the association between ABSI and 20-meters SRT using methods such as one-way analysis of variance (ANOVA) and linear regression analysis.

**Results:**

Among adolescents aged 6–17 years in rural China, the ABSI was (0.069 ± 0.013) and the 20-m SRT was (29.69 ± 17.16) laps. Overall, there were statistically significant differences in the interquartile ranges of the 20-m SRT across the 6–8, 9–11, 12–14, and 15–17 age groups (*F* = 33.841, 14.916, 13.858, 7.715; *P* < 0.001). Curvilinear regression analysis showed an inverted *U*-shaped relationship between ABSI and 20-m SRT. Overall, in the 6–8 years, 9–11 years, 12–14 years, and 15–17 years age groups, the 20-m SRT was highest when the ABSI was 0.056, 0.070, 0.094, and 0.054, respectively, reaching 18.673, 26.226, 38.328, and 40.499 laps, respectively.

**Conclusions:**

There is an inverted *U*-shaped relationship between ABSI and 20-meter SRT among adolescents in rural China. Maintaining a reasonable ABSI may on CRF.

## 1 Introduction

As lifestyles and environmental conditions continue to change, the incidence of CRF among adolescents is on the decline, and it has become a public health issue faced by countries around the world (1). A survey of 76,000 adolescents in the Mid-Atlantic region shows that overall CRF among adolescents is on a downward trend (2). Other studies have shown that a longitudinal study of physical fitness among adolescents in 19 high- and upper-middle-income countries found a marked decline in adolescent physical fitness between 1981 and 2014, consistent with widespread global concerns about the physical health of adolescents (3). This indicates that a decline in adolescents' physical fitness is a widespread phenomenon in developed countries. Similarly, adolescents in developing countries are no exception. An analysis of physical fitness among 7- to 18-year-olds in China's Xinjiang region from 1985 to 2019 showed that while physical fitness improved between 1985 and 1995, it deteriorated significantly between 1995 and 2005. Although the rate of decline slowed after 2005, most indicators continued to decline between 2014 and 2019, severely impacting adolescents' physical and mental health as well as their future health in adulthood (4). Another meta-analysis of Chinese adolescents showed that the 20-m SRT score was 34.67 laps and the maximum oxygen uptake (VO_2max_) was 46.47 ml/kg/min, which falls within the healthy range but is not at the optimal level (5). Another study also found that CRF in adolescents is associated with academic performance, mental health, and various diseases (6). Research shows that adolescents' CRF levels are significantly positively correlated with their math and English grades, possibly because higher CRF levels are associated with improved executive function and enhanced memory function in the brain (7). At the same time, relatively little research has examined with urban adolescents, rural adolescents exhibit a more pronounced decline in CRF. This finding warrants attention; urban adolescents exhibit a more significant decline in CRF. However, most previous studies have focused on adolescents in urban areas. At the same time, relatively little research has examined rural adolescents, who constitute a significant proportion of the population (8). Previous studies have shown that, compared with urban adolescents, rural adolescents exhibit a more pronounced decline in CRF. This finding warrants attention; rural adolescents exhibit a more significant decline in CRF. Therefore, effectively analyzing the factors that influence adolescents' CRF will help improve their CRF levels and their physical and mental health.

Direct and indirect measures the in recent years, there has been a gradual increase in the use of indirect testing methods to assess CRF in adolescents, primarily the 20-m SRT and the Cooper 12-min run, which are used to estimate their CRF levels (9). Indirect testing methods are widely used they impose relatively low demands on equipment and test administrators. The 20-m shuttle run test (20-m SRT) was first proposed by (10) in 1984 and is currently used in more than 50 countries worldwide; it is effective in assessing CRF in adolescents and has good reliability and validity (10). Similarly, in recent years, there has been a growing number of studies using the 20-m SRT method to assess CRF in Chinese adolescents, and these studies have gained widespread recognition and acceptance (11).

As a new indicator of body composition, the body shape index (ABSI) is significantly associated with multiple health indicators (12). In the UK Health and Lifestyle Survey, a one-standard-deviation increase in the ABSI was associated with a 13% increase in mortality risk and demonstrated superior predictive ability compared with traditional measures of abdominal obesity, such as waist circumference and waist-to-height ratio (13). Body composition indicators, the ABSI demonstrated superior predictive power for all-cause mortality risk. Research also indicates that there is a significant association between the ABSI and mental health; the ABSI is effective in identifying abnormal psychological behaviors in adolescents and plays a positive role in preventing the onset of mental health issues (14). However, it is worth noting that previous studies have primarily focused on the association between ABSI in adolescents or adults and physical illness and mental health. At the same time, there has been relatively little research on the association between ABSI and CRF (15). Previous studies have shown a significant association between the ABSI and muscle strength, with the two exhibiting an inverted *U*-shaped relationship (16). Additionally, other studies have found a significant association between the ABSI and grip strength (17). However, the findings regarding the association between the ABSI and health are not entirely consistent. A study of adolescents found that the ABSI was not effective and prediabetes, whereas BMI and waist circumference performed better (18). The association between the ABSI and health requires further validation and analysis. Notably, no significant association has yet been identified between the ABSI and CRF among adolescents in rural China. As a core indicator of adolescents' physical fitness, CRF is significantly associated with adolescents' physical and mental health; therefore, analyzing its influencing factors is of practical importance.

As a developing country, China has significant disparities between its urban and rural areas (19). Previous studies have shown significant differences between urban and rural adolescents in China across multiple indicators, including body composition, physical fitness, and motor skills (20). The overall physical fitness of adolescents in rural China is lower than that of their urban counterparts (21). It is also worth noting that in recent years, as the economy has continued to develop, the rate of obesity among adolescents in rural China has been rising faster than in urban areas, posing a serious threat to the healthy development of rural adolescents, with particularly significant implications for CRF (22). To better promote the development and improvement of physical fitness among adolescents in rural China, this study examined 18,948 adolescents in rural areas to analyze the relationship between the ABSI and the 20-m shuttle run test (SRT), measures a measure of physical fitness. The aim is to provide guidance and support for enhancing the physical fitness of adolescents in rural China.

## 2 Methods

### 2.1 Participants

This study employed a multi-stage random cluster sampling method to select participants. First, based on China's geographical divisions, two rural areas were selected from each of the southern, northern, western, and eastern regions as the sampling areas for participants. From the rural schools in each region, four elementary schools and four middle schools were selected for evaluation. Within each grade level, classes were used as the sampling units, and one teaching class was randomly cluster-sampled from each grade level to serve as the test class for this study. Ultimately, this study conducted a cross-sectional assessment of 20,104 adolescents aged 6–17 years across 384 school classes. After the assessment, 1,156 invalid questionnaires were excluded, leaving 18,948 valid questionnaires and a response rate of 94.25%. The number of people in each age group is shown in Table 1. Inclusion criteria for this study were: currently enrolled students who provided written informed consent from both parents and the student, and who voluntarily agreed to participate in the assessment. Exclusion criteria included: refusal by parents or the student to participate, or the presence of physical disabilities or mental health conditions. The primary reasons for excluding invalid data or questionnaires after data collection included missing key demographic information, damaged questionnaires, or illegible responses.

**Table 1:** Distribution of adolescents across different age groups in rural China.

Age (years)	Boys	Girls	Total
6 years	1,031 (53.1)	909 (46.9)	1,940
7 years	697 (52.6)	628 (47.4)	1,325
8 years	790 (49.0)	821 (51.0)	1,611
9 years	868 (52.7)	780 (47.3)	1,648
10 years	869 (53.5)	754 (46.5)	1,623
11 years	790 (53.1)	698 (46.9)	1,488
12 years	802 (53.3)	704 (46.7)	1,506
13 years	751 (51.7)	701 (48.3)	1,452
14 years	855 (50.9)	825 (49.1)	1,680
15 years	869 (48.9)	907 (51.1)	1,776
16 years	760 (48.0)	822 (52.0)	1,582
17 years	640 (48.6)	677 (51.4)	1,317
6–17 years	9,722 (51.3)	9,226 (48.7)	18,948

This study was conducted in accordance with the principles of the Declaration of Helsinki. Written assent was obtained from parents/guardians. The participants also signed up for the study on their own. The study was approved by the Biomedical Research Ethics Committee of Gannan Medical University (2024724).

### 2.2 A body shape index

The ABSI is calculated using a fixed formula based on BMI and waist circumference. The specific formula is: ABSI = Waist Circumference (m)/(BMI^∧^(2/3) × Height^∧^(1/2)). BMI is calculated as weight (kg) divided by height (m)^2^ (23). In this study, the assessment criteria for height, weight, and waist circumference were based on the instruments and methods specified in the China National Survey on Students' Constitution and Health (CNSSCH) (24). Height and waist circumference are measured to the nearest 0.1 cm, and weight is measured to the nearest 0.1 kg.

### 2.3 Cardiorespiratory fitness

In this study, CRF was assessed using the 20-m SRT, a test that is currently widely used internationally (25). The 20-m SRT is a commonly used method for assessing physical fitness in adolescents. Because the test is simple and can be administered on a large scale, it is currently used in more than 50 countries worldwide (26). At the same time, it has been widely adopted in China in recent years and has proven effective in assessing CRF among Chinese adolescents (27). The 20-m SRT assessment must be conducted on a flat school basketball court or a track-and-field surface. Participants must complete the assessment under the supervision of staff. Before the assessment, participants are required to perform a warm-up to prevent sports injuries. The 20-m SRT requires participants to assess background music. Participants begin running upon hearing a “beep” and must reach the opposite end line by the time the next beep sounds; this is recorded as 1 lap (20 m). The initial speed for the 20-m SRT is 8.0 km/h, increasing to 9.0 km/h in the second minute, rising by 0.5 km/h per minute. The entire assessment consists of 127 laps (28). The more laps participants run, the higher their CRF level.

### 2.4 Covariates

The covariates in this study include father's occupation, mother's occupation, father's education, mother's education, and household income. Participants self-reported these covariates based on their actual circumstances by completing a questionnaire. Father's occupation and mother's occupation are categorized as civil servant, laborer, office worker, business owner, farmer, or other. Father's education and mother's education were categorized as elementary school, middle school, high school, or college or higher. Household income was categorized as less than 2,000 yuan/month, 2,001–5,000 yuan/month, 5,001–8,000 yuan/month, or more than 8,000 yuan/month.

### 2.5 Statistical analysis

The results of this study are presented using both categorical and continuous variables. Categorical variables are expressed as percentages. Continuous variables are expressed as means and standard deviations. After stratification by age and sex, the ABSI was divided into quartiles, resulting in four groups: ABSI <25th percentile (A), 25th ≤ ABSI <50th percentile (B), 50th ≤ ABSI <75th percentile (C), and ABSI ≥ 75th percentile (D). Comparisons of 20-m SRT scores between ABSI quartile groups were performed using one-way analysis of variance (ANOVA), with LSD tests used for intergroup comparisons. The association between ABSI and 20-m SRT was analyzed using curve regression analysis. Curve regression analysis was conducted with 20-m SRT as the dependent variable and ABSI as the independent variable to derive the fitted regression equation. The β_1_ and β_2_ coefficients of the fitted equation, along with their 95% confidence intervals, were reported, along with the *R*^2^ value and *P*-value. Based on this, a quadratic curve was plotted for the relationship between ABSI and the 20-m SRT. SPSS 25.0 software was used for statistical analysis in this study.

## 3 Results

This study conducted a cross-sectional assessment of height, weight, waist circumference, and 20-m SRT among 18,948 adolescents aged 6–17 years in rural China, including 9,722 boys and 9,226 girls. The mean ABSI in this study was (0.069 ± 0.013), and the mean 20-m SRT was (29.69 ± 17.16).

The results of this study show that, overall, there were statistically significant differences between different age groups in terms of height, weight, BMI, waist circumference (WC), abdominal stiffness index (ABSI), and 20-m SRT (χ^2^ = 17,089.686, 8,343.326, 1,411.826, 1974.275, 2030.019, 2230.200, *P* < 0.001). The same trend was observed among both male and female participants. Specific results are presented in Table 2.

**Table 2:** One-way analysis of variance on basic conditions of adolescents in rural China (*M* ± *SD*).

Sex/Age Group	*N*	Height	Weight	Body mass index	Waist circumference	ABSI	20-m SRT
Boys							
6–8 years	2,518	132.07 ± 9.05	30.11 ± 7.62	17.10 ± 3.14	57.50 ± 11.17	0.078 ± 0.016	17.92 ± 8.65
9–11 years	2,527	149.54 ± 10.02	42.53 ± 10.91	18.82 ± 3.53	65.92 ± 11.09	0.072 ± 0.012	27.54 ± 12.45
12–14 years	2,408	168.53 ± 8.41	57.08 ± 12.32	19.98 ± 3.57	70.91 ± 10.4	0.064 ± 0.01	43.77 ± 18.39
15–17 years	2,269	173.67 ± 6.28	63.22 ± 10.98	20.91 ± 3.17	74.04 ± 9.54	0.062 ± 0.008	48.95 ± 19.88
*F*-value		11,835.576	4,790.095	577.583	1,126.263	903.145	2,113.67
*P*-value		<0.001	<0.001	<0.001	<0.001	<0.001	<0.001
Girls							
6–8 years	2,358	130.79 ± 8.66	27.90 ± 6.29	16.18 ± 2.58	54.97 ± 9.05	0.079 ± 0.015	17.39 ± 7.87
9–11 years	2,232	149.65 ± 9.12	40.10 ± 9.44	17.75 ± 3.15	62.75 ± 9.18	0.072 ± 0.012	23.68 ± 10.74
12–14 years	2,230	160.85 ± 5.94	50.87 ± 8.28	19.63 ± 2.81	66.33 ± 8.11	0.064 ± 0.009	28.06 ± 14.10
15–17 years	2,406	161.91 ± 5.53	52.50 ± 7.19	20.02 ± 2.47	67.18 ± 7.38	0.063 ± 0.007	31.16 ± 11.38
*F*-value		8,775.801	4,921.007	972.022	1,019.577	1,162.359	671.114
*P*-value		<0.001	<0.001	<0.001	<0.001	<0.001	<0.001
Total							
6–8 years	4,876	131.45 ± 8.89	29.04 ± 7.10	16.66 ± 2.92	56.27 ± 10.28	0.079 ± 0.015	17.66 ± 8.28
9–11 years	4,759	149.59 ± 9.61	41.39 ± 10.31	18.32 ± 3.40	64.43 ± 10.36	0.072 ± 0.012	25.73 ± 11.83
12–14 years	4,638	164.84 ± 8.28	54.09 ± 11.02	19.81 ± 3.23	68.70 ± 9.64	0.064 ± 0.01	36.22 ± 18.24
15–17 years	4,675	167.62 ± 8.33	57.7 ± 10.67	20.45 ± 2.87	70.51 ± 9.16	0.062 ± 0.007	39.80 ± 18.37
*F*-value		17,089.686	8,343.326	1,411.826	1,974.275	2,030.019	2,230.200
*P*-value		<0.001	<0.001	<0.001	<0.001	<0.001	<0.001

*N*, number; M, Mean; SD, standard deviation; ABSI, a body shape index.

The ABSI scores were divided into quartiles by gender and age, and a one-way ANOVA was conducted between groups after stratification by gender and age group. Overall, the results showed that in the 6–8, 9–11, 12–14, and 15–17 years age groups, the between-group differences in 20-m SRT were all statistically significant (*F* = 33.841, 14.916, 13.858, 7.715, *P* < 0.001). The results of the comparative analysis, stratified by gender, are shown in Table 3.

**Table 3:** A univariate comparison of the ABSI and 20-m SRT among adolescents in rural China.

Age (years)	ABSI<25th (A)		25th ≤ ABSI <50th (B)		50th ≤ ABSI<75th (C)		ABSI ≥75th (D)		*F*-value	*P*-value	LSD^#^					
	*N*	*M* (*SD*)	*N*	*M* (*SD*)	*N*	*M* (*SD*)	*N*	*M* (*SD*)			A/B	A/C	A/D	B/C	B/D	C/D
Boys																
6–8 years	286	17.60 ± 8.33	184	18.87 ± 9.21	633	20.14 ± 8.94	1,415	16.86 ± 8.30	22.398	<0.001		^#^			^#^	^#^
9–11 years	376	27.59 ± 12.79	497	28.09 ± 13.29	964	28.21 ± 12.7	690	26.18 ± 11.12	4.031	0.007					^#^	^#^
12–14 years	851	44.15 ± 18.62	762	44.15 ± 17.91	605	44.33 ± 18.06	190	38.83 ± 19.72	5.000	0.002			^#^		^#^	^#^
15–17 years	1,036	49.41 ± 20.17	820	49.52 ± 19.80	338	47.57 ± 19.75	75	42.71 ± 15.87	3.430	0.016			^#^		^#^	
Girls																
6–8 years	218	17.44 ± 7.63	157	18.92 ± 8.66	566	18.76 ± 8.02	1,417	16.66 ± 7.66	11.942	<0.001		^#^			^#^	^#^
9–11 years	309	20.10 ± 12.24	497	24.65 ± 11.09	749	25.21 ± 10.13	677	22.91 ± 9.94	19.513	<0.001	^#^	^#^	^#^		^#^	^#^
12–14 years	759	23.36 ± 13.71	870	29.81 ± 13.11	496	32.14 ± 14.08	105	28.15 ± 15.58	49.443	<0.001	^#^	^#^	^#^	^#^		^#^
15–17 years	924	31.19 ± 11.18	1,018	31.92 ± 11.67	399	30.08 ± 11.02	65	25.52 ± 9.71	8.106	<0.001			^#^	^#^	^#^	^#^
Total																
6–8 years	504	17.53 ± 8.02	341	18.89 ± 8.95	1,199	19.49 ± 8.54	2,832	16.76 ± 7.99	33.841	<0.001	^#^	^#^			^#^	^#^
9–11 years	685	24.21 ± 13.08	994	26.37 ± 12.35	1,713	26.90 ± 11.73	1,367	24.56 ± 10.67	14.916	<0.001	^#^	^#^			^#^	^#^
12–14 years	1,610	34.35 ± 19.48	1,632	36.51 ± 17.1	1,101	38.84 ± 17.47	295	35.03 ± 19.03	13.858	<0.001	^#^	^#^		^#^		^#^
15–17 years	1,960	40.82 ± 18.88	1,838	39.77 ± 18.08	737	38.10 ± 17.90	140	34.73 ± 15.86	7.715	<0.001		^#^	^#^	^#^	^#^	^#^

^#^*P* < 0.05; N, numbers; M, Mean; SD, standard deviation; ABSI, a body shape index.

After performing a regression analysis, the following regression equation was obtained:

Boys:

6–8 years 20-m SRT = −1,736.538 × *A*^2^ + 214.760 × *A* + 12.177

β_1_ = 214.760 (95% CI: 114.144 to 315.376), β_2_ = −1,736.538 (95% *CI*: −2,385.117 to −1,087.959), *R*^2^ = 0.019

9–11 years 20-m SRT = −2,838.542 × *A*^2^ + 360.654 × *A* + 16.683

β_1_ = 360.654 (95%CI: 127.325 to 593.984), β_2_ = −2,838.542 (95% CI: −4,412.813 to −1,264.271), *R*^2^ = 0.008

12–14 years 20-m SRT = −830.674 × *A*^2^ + 46.689 × *A* + 44.289

β_1_ = 46.689 (95% CI: −219.005 to 312.384), β_2_ = −830.674 (95% CI: −2,618.908 to 957.561), *R*^2^ = 0.002

15–17 years 20-m SRT = −11,350.943 × *A*^2^ + 1,424.711 × *A* + 4.943

β_1_ = 1,424.711 (95% CI: 666.410 to 2,183.013), β_2_ = −11,350.943 (95% CI: −17,182.415 to −5,519.470), *R*^2^ = 0.007

Girls:

6–8 years 20-m SRT = −838.469 × *A*^2^ + 66.890 × *A* + 17.533

β_1_ = 66.890 (95% CI: −60.955 to 194.736), β_2_ = −838.469 (95% CI: −1,662.484 to −14.454), *R*^2^ = 0.014

9–11 years 20-m SRT = −3,921.280 × *A*^2^ + 593.023 × *A* + 1.899

β_1_ = 593.023 (95% CI: 371.805 to 814.240), β_2_ = −3,921.280 (95% CI: −5,346.536 to −2,496.023), *R*^2^ = 0.013

12–14 years 20-m SRT = −7,894.209 × *A*^2^ + 1,386.620 × *A* – 27.659

β_1_ = 1,386.620 (95% CI: 1,050.156 to 1,723.083), β_2_ = −7,894.209 (95% CI: −10,294.987 to −5,493.432), *R*^2^ = 0.054

15–17 years 20-m SRT = −6,619.566 × *A*^2^ + 797.113 × *A* + 7.532

β_1_ = 797.113 (95% CI: 294.355 to 1,299.871), β_2_ = −6,619.566 (95% CI: −10,425.174 to −2,813.959), *R*^2^ = 0.007

Total:

6–8 years 20-m SRT = −1,409.886 × *A*^2^ + 159.726 × *A* + 14.150

β_1_ = 159.726 (95% CI: 81.471 to 237.980), β_2_ = −1,409.886 (95% CI: −1,914.471 to −905.301), *R*^2^ = 0.016

9–11 years 20-m SRT = −3,182.446 × A^2^ + 439.756 × A + 11.037

β_1_ = 439.756 (95% CI: 277.029 to 602.482), β_2_ = −3,182.446 (95% CI: −4,255.809 to −2,109.083), *R*^2^ = 0.008

12–14 years 20-m SRT = −2,259.265 × A^2^ + 420.710 × A + 18.744

β_1_ = 420.710 (95% CI: 196.806 to 644.614), β_2_ = −2,259.265 (95% CI: −3,801.776 to −716.754), *R*^2^ = 0.005

15–17 years 20-m SRT = −5,243.758 × *A*^2^ + 559.724 × *A* + 25.565

β_1_ = 559.724 (95% CI: 30.705 to 1,088.744), β_2_ = −5,243.758 (95% CI: −9,283.801 to −1,203.715), *R*^2^ = 0.004

A stands for ABSI.

**Figure 1:**
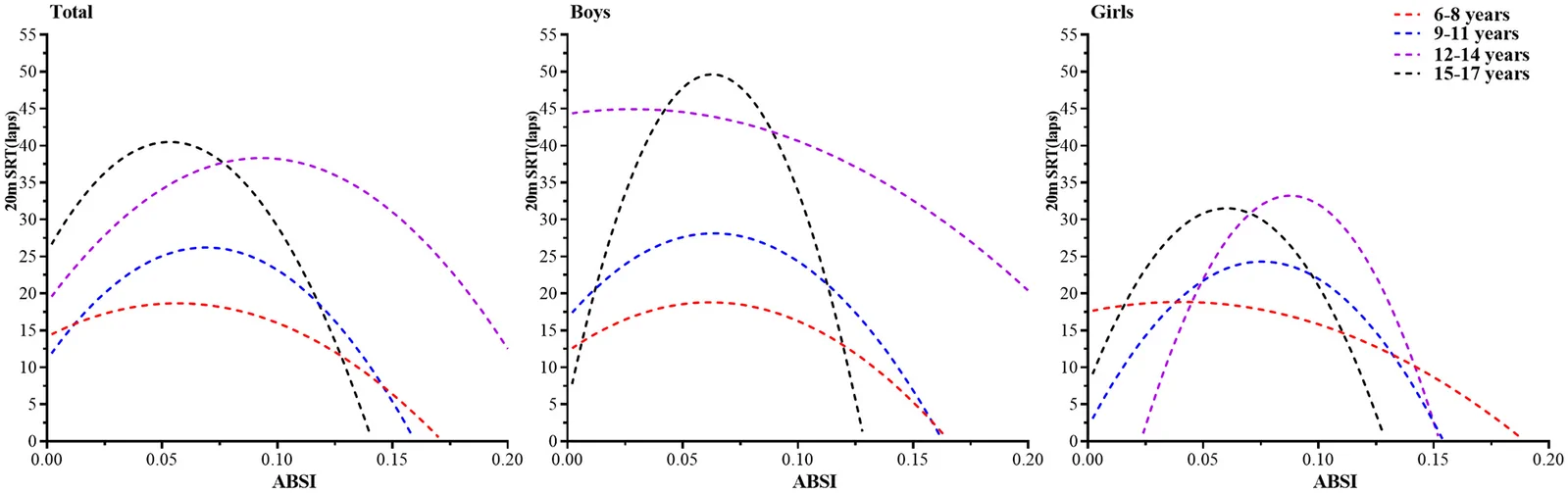
Regression curves for ABSI and 20-m SRT among adolescents in rural China.

Based on the equation, regression curves were plotted for ABSI and 20-m SRT. Overall, an inverted *U*-shaped relationship was observed between ABSI and 20-m SRT. Overall, in the 6–8, 9–11, 12–14, and 15–17 age groups, when ABSI was 0.056, 0.070, 0.094, and 0.054, respectively, the 20-m SRT levels were highest, reaching 18.673, 26.226, 38.328, and 40.499 laps, respectively. See Figure 1 for specific trends.

## 4 Discussion

As the economy and living standards continue to evolve, significant changes have occurred in the body composition of adolescents in rural China. Studies show that the prevalence of overweight and obesity among adolescents in rural China is higher than in urban areas, posing serious negative impacts on their physical and mental health. Furthermore, the rate of increase in overweight and obesity is higher in rural areas than in urban areas, and this issue warrants sufficient attention and priority (29). As a new indicator that comprehensively reflects body composition in adolescents, the ABSI has garnered widespread attention and recognition in recent years. Research indicates that, compared with conventional body composition indicators such as BMI and waist circumference, the ABSI—by incorporating waist circumference—provides a more comprehensive and accurate reflection of body fat content in adolescents, making it a valuable tool for assessing adolescent health (30). The results of this study indicate that ABSI among adolescents in rural China declines gradually with increasing age. There are multiple factors contributing to this finding. First, the adolescents surveyed in this study were aged 6–17; during this stage, adolescents are at the peak of puberty, characterized by rapid height growth, a gradual decrease in body fat, and a continuous increase in muscle mass, all of which contribute to the downward trend in ABSI (31). Previous studies have shown that adolescents' ABSI tends to decline during puberty, consistent with the findings of this study (32). Second, during puberty, as hormone levels continue to rise—with increased secretion of testosterone in boys and estrogen in girls—bones grow rapidly. Combined with a steady increase in physical activity, this leads to a reduction in body fat among adolescents, which is another key factor contributing to the gradual decline in ABSI (16). Research shows that during adolescence, teenagers experience rapid increases in height, decreases in body fat, and significant changes in body composition (33).

In addition to significant changes in body composition, adolescents also experience substantial changes in their physical fitness levels. The results of this study indicate that the 20-m SRT, which reflects adolescents' CRF, shows an upward trend with increasing age, consistent with the findings of numerous studies (11). Contributing to the gradual increase in the 20-m SRT among adolescents in rural areas in this study. First, as adolescents continue to develop physically, their muscle strength steadily increases; this increase in muscle strength during the 20-m SRT test contributes to improved performance. Research indicates that muscle strength in adolescents is significantly and improved CRF (34). Second, as adolescence progresses, adolescents' hormone levels continue to rise, leading to increased levels of physical activity; this is another key factor contributing to the gradual increase in 20-m SRT observed in this study. Previous research has found a positive correlation between adolescents' physical activity levels and CRF, such that adolescents with higher physical activity tend to have higher CRF. In addition, body weight is a significant factor affecting the 20-m SRT in adolescents. However, as puberty sets in, adolescents experience a decrease in body fat, accompanied by continuous improvements in height, lung function, and cardiac function—another key reason for the steady increase in the 20-m SRT (35).

The results of this study indicate an inverted *U*-shaped relationship between the ABSI and the 20-m SRT—a measure of CRF—among adolescents in rural China: both extremely low and extremely high ABSI scores are associated with lower CRF. This suggests that adolescents in rural China should maintain a reasonable ABSI level to better promote CRF. Contributing to the inverted *U*-shaped relationship between ABSI and the 20-m SRT. First, there is a non-linear relationship between body composition and CRF. Lower ABSI levels indicate lower body weight, which leads to insufficient muscle strength and relatively weaker aerobic energy production, resulting in poorer 20-m SRT performance. Second, the physical changes that occur during the growth and development phase lead to a rapid increase in height and during puberty. This increase in height results in a longer stride, which facilitates better performance on the 20-m SRT assessment. Third, there is a non-linear relationship between physical fitness and CRF. With the onset of adolescence, adolescents' physical fitness tends to increase, primarily due to a sustained rise in their physical activity. Research indicates that increased physical activity among adolescents leads to the continued strengthening of the cardiopulmonary system, enhancing the body's ability to transport and utilize oxygen, thereby further improving CRF (36). Other studies have shown that adolescents' physical activity levels are positively correlated with the 20-m SRT. Increasing physical activity levels can effectively improve physical fitness, including explosive power, muscle strength, and muscular endurance, thereby effectively enhancing overall physical fitness (37). Fourth, metabolic and endocrine changes during adolescence are also key factors contributing to changes in CRF. Studies have shown that the sharp rise in growth hormone levels during puberty further enhances aerobic capacity. The underlying mechanism may be that growth hormone secretion in the number and volume of mitochondria, thereby improving the efficiency of aerobic oxidation and enhancing CRF levels (38). In addition, an increase in ABSI is associated with increased body fat, and increased body fat can lead to heightened systemic inflammation, which negatively impacts CRF levels. Studies have shown that systemic inflammation can cause persistent elevations in inflammatory factors, damage vascular endothelial cells, reduce vasodilation capacity, increase peripheral resistance, impair blood flow, and reduce the blood's ability to transport oxygen, thereby affecting CRF levels (39).

To our knowledge, this study is the first to examine the association between the ABSI and the 20-m SRT using a large sample from rural areas across China. The findings of this study may provide valuable insights and guidance for improving adolescents' physical fitness levels in rural China. However, this study also has certain limitations. First, as a cross-sectional study, it can only analyze the association between ABSI and the 20-m SRT, but cannot determine a causal relationship between the two. Future prospective cohort studies should be conducted to analyze the causal relationship between these factors. Secondly, this study used the 20-m SRT to indirectly assess adolescents' CRF levels, which may have introduced some bias compared to actual results. Future studies should employ more accurate assessment methods to measure adolescents' CRF levels, thereby enhancing the validity of the findings. Finally, the limited number of covariates included in this study's analysis of the association between ABSI and the 20-m SRT may have influenced the results. Future studies should incorporate additional covariates, such as sleep quality, pubertal development, dietary habits, school physical education policies, and physical activity, to more accurately analyze the relationship between the two variables.

## 5 Conclusions

There is an inverted *U*-shaped relationship between the ABSI and 20-m SRT among adolescents in rural China, and this relationship holds for both boys and girls. Overall, when the ABSI ranges from 0.054 to 0.094, the 20-m SRT is relatively high. Effectively controlling ABSI levels may CRF levels among adolescents in rural China.

## Data Availability

The raw data supporting the conclusions of this article will be made available by the authors, without undue reservation.
